# Primigravida with Bernard-Soulier Syndrome: a case report

**DOI:** 10.1186/s13104-015-1145-5

**Published:** 2015-05-01

**Authors:** Marina Barguil Macêdo, Janaína de Moraes Machado Brito, Plínio da Silva Macêdo, José Araújo Brito

**Affiliations:** Federal University of Piauí, Dona Evangelina Rosa Maternity Hospital, Av. Higino Cunha, 1552, Ilhotas, Teresina, PI Zip Code 64014-220 Brazil; Integral Differential Faculty/Devry, Dona Evangelina Rosa Maternity Hospital, Av. Higino Cunha, 1552, Ilhotas, Teresina, PI Zip Code 64014-220 Brazil; Department of Pathology and Dentistry Clinic, Federal University of Piauí (UFPI), Campus Universitário Ministro Petrônio Portela, Ininga, Teresina, PI Zip Code 64049-550 Brazil; Department of Maternal-Fetal Medicine, Federal University of Piauí, Dona Evangelina Rosa Maternity Hospital, Av. Higino Cunha, 1552, Ilhotas, Teresina, PI Zip Code 64014-220 Brazil

**Keywords:** Bernard-Soulier syndrome, Blood coagulation disorders, Blood platelet disorders, Platelet transfusion, Pregnancy complications, hematologic

## Abstract

**Background:**

Bernard-Soulier Syndrome is a rare congenital bleeding disorder, mainly inherited in an autosomal recessive pattern. It is characterized by a genetic defect on one of the four genes encoding the subunits of the transmembrane protein complex GPIb-V-IX, physiologically expressed only in platelets. The exact phenotype varies widely from individual to individual depending on the particular mutation presented. Currently, there is no consensus about ideal management of affected pregnant women, in face of the scarcity of cases.

**Case presentation:**

We report on a 28-year-old Black Brazilian primigravida who was referred to our maternity hospital, a tertiary care center, for decision about the most adequate mode of delivery. She was admitted with a platelet count of 43.000 plt/μL, and hemoglobin of 13.6 g/dL. Platelet transfusion was regarded as a necessary prophylactic measure prior to delivery. Ten units of random donor platelets were administered on the course of three days, after which the patient was submitted to an elective cesarean section delivery under general anesthesia at 40 weeks of gestational age. A healthy male baby with a normal birthweight of 3.615 kg was delivered. After the delivery, the mother’s state continued being assessed daily, with special attention taken to lochia and surgical wound healing. At one week postpartum, a complete blood count revealed a platelet count of 41.000 plt/μL, and hemoglobin of 13.3 g/dL. As there were no signs of neither evident nor occult hemorrhage, and surgical wound was healing accordingly, the patient was discharged, after being oriented about bleeding preventive measures.

**Conclusion:**

The peripartum period is regarded as the most crucial moment of pregnancy in women with Bernard-Soulier Syndrome, hence the importance of a judiciously planned mode of delivery, and of careful prophylaxis against bleeding beforehand. Furthermore, absence of complications during the peripartum period does not predict how the woman will do subsequently. Strict vigilance is warranted at least until six weeks postpartum, due to the virtual risk of secondary postpartum hemorrhage.

## Background

Bernard-Soulier Syndrome (BSS) is a rare congenital bleeding disorder, mainly inherited in an autosomal recessive pattern [1]. On molecular terms, it is characterized by a genetic defect on one of the four genes encoding the subunits of the transmembrane protein complex GPIb-V-IX, physiologically expressed only in platelets. The mutated complex is unable to perform its primary function, namely, to normally bind von Willebrand factor [1-3]. As a result, a constellation of symptoms may arise from the abnormally functioning coagulation cascade – from recurrent spontaneous and self-limiting epistaxis to life-threatening hemorrhages – but the exact phenotype varies widely from individual to individual depending on the particular mutation presented [2]. The affected individual may, as well, remain asymptomatic or oligosymptomatic for a long time, and receive the diagnosis by the means of a routine laboratory tests disclosing a low count of very large platelets, accompanied by a prolonged bleeding time and normal clotting factors level. Failure to agglutinate with ristocetin (even after the addition of normal plasma), the hallmark of the syndrome, must be proved in order to rule out other disorders manifesting with macrothrombocytopenia [1,2]. The importance of appropriately diagnosing the disease is to prevent major bleedings by correctly orienting the patient how to avoid situations that may be potentially dangerous, such as contact sports, and how to proceed when a bleeding does occur.

Pregnancy in a woman with this uncommon syndrome must be carefully monitored, once it is a singular situation in which the patient is especially susceptible to bleeding episodes, which could translate into maternal and neonatal unfavorable outcomes [4]. Since it was first described by Jean Bernard and Jean-Pierre Soulier, in 1948, more than a hundred individuals were given the diagnosis worldwide, but only twenty-one women with the syndrome had their pregnancies reported on indexed journals up until now [3,5]. Since the body of evidence on this field is still limited, with no established consensus on the best way to approach pregnancy in those patients, it is particularly interesting to share experiences on how to manage the intrapartum and the postpartum periods in BSS, which motivated us to report the successful pregnancy outcome of a 28-year-old Brazilian primigravida with BSS. The patient signed an informed consent agreeing to have her case published. To our knowledge, this is only the second case of a South American pregnant woman with BSS, the first one being published two decades ago; and the first case of a woman of Black ethnicity, the other ones being Caucasian, Asian, or Latin American in origin [3].

## Case presentation

A 28-year-old Black Brazilian woman, gravida 1, para 0, was referred to our maternity hospital for close monitoring at 39 weeks 4 days of gestational age (calculated by first trimester obstetric ultrasonography), after her latest prenatal visit has revealed a low platelet count (PC) of 65.000 plt/μL. She had received the diagnosis of Bernard-Soulier syndrome ten years ago, suspected by the finding of thrombocytopenia with giant platelets on a routine complete blood count (CBC), and confirmed by the finding of failed ristocetin-induced platelet aggregation, even after the addition of normal plasma. Prior to the diagnosis, she complained of had suffering, during her childhood, from several episodes of epistaxis while sneezing, as well as having experienced gingival bleeding after minimal trauma while toothbrushing. None of the episodes required medical intervention. However, she affirmed having received platelet transfusion as prophylaxis against hemorrhage before undergoing teeth extraction. She denied menorrhagia or developing significant hematomata after accidental bruises.

The patient was referred to our maternity hospital, the only tertiary care center of the region, for assessment of the best mode of delivery in face of her condition. She was admitted to our wards and a CBC was performed, confirming a low PC of only 43.000 plt/μL, a hemoglobin (Hb) of 13.6 g/dL, and a hematocrit (Hct) of 39%. Activated clotting time was 6 minutes, clot retraction was normal, prothrombin time was 13.2 seconds, International Normalized Ratio (INR) was 1.3, and activated partial thromboplastin time was 31.9 seconds. An obstetric ultrasound revealed a placental grading (Grannum classification) of II, a normal amniotic fluid index of 13.1 cm, and no signs of growth restriction or fetal distress. Physical examination was unremarkable. The patient denied the occurrence of any bleeding episode demanding intervention during the antenatal period, reporting only minor gingival bleeding after tooth brushing. Her baseline PC varied from 75.000 plt/μL to 120.000 plt/μL.

Platelet transfusion was regarded as a necessary prophylactic measure prior to delivery. Ten units of random donor platelets were administered on the course of three days, after which the patient was submitted to an elective cesarean section delivery under general anesthesia, at gestational age of 40 weeks. A healthy male baby with a normal birthweight of 3.615 kg was delivered. His 1-minute and 5-minute Apgar scores were both 8.

After the delivery, the mother’s state continued being assessed daily, with special attention taken to lochia and surgical wound healing. At one week postpartum, a CBC revealed a PC of 41.000 plt/μL, Hb of 13.3 g/dL, and Hct of 39%. Since there were no signs of neither evident nor occult hemorrhage, and the surgical wound was healing accordingly, the patient was discharged.

## Discussion

Bernard-Soulier syndrome is a platelet disorder inherited most often as a recessive trait. As so, consanguinity must be sought whenever a proband is identified [1]. On a systematic review covering the topic, one third of the analyzed women reported being born from consanguineous couples [2]. When questioned about it, our patient revealed that her parents were cousins. She also mentioned that she had three sisters and two brothers, but none was diagnosed with clotting problems.

Owing to the diverse phenotypic presentation of the syndrome and its nonspecific symptoms, age at diagnosis differs widely from patient to patient. Affected females are usually diagnosed after menarche, with a mean age of 19 years reported in a recent review [2]. Excessive bleeding during menstruation is one of the most frequent and prominent symptoms, although not constant. Our patient presented only minor nasal and gingival hemorrhage episodes, and no menorrhagia, which concurred to the delay to seek specialized medical advice until she was eighteen.

Pregnancy in BSS is also associated with a variable course. The outcome varies among different patients and even for the same patient in different pregnancies [2,4]. It may evolve in a pattern similar to that of women who do not have the syndrome, or it may be complicated by maternal and fetal morbidity of various severity degrees [1]. For the mother, the increased risk of bleeding warrants the need of thorough prenatal and postnatal care. For the fetus, placental transfer of maternal antiplatelet antibodies poses a risk of alloimmune neonatal thrombocytopenia [6], for which a readily available treatment is essential. In face of these considerations, delivery at a tertiary center is advised whenever possible [2,7], inasmuch as complications during or immediately after labor may require prompt intervention.

Indeed, the peripartum period is regarded as the most crucial moment of pregnancy in women with BSS [4,8], hence the importance of a judiciously planned mode of delivery, and of careful prophylaxis against bleeding beforehand. Our patient received platelet transfusion as prophylaxis, once this is regarded as the most efficient and safest measure prior to a surgery in individuals with inherited platelet disorders [9,10].

Cesarean section under general anesthesia was indicated for our patient, since the fetus had already reached term, and an unremarkable antenatal period is not reassuring for the absence of bleeding at late pregnancy [10], which may be disastrous for both mother and fetus. There is no statistically significant difference in outcomes of pregnancy from women delivering vaginally or by c-section, and there is currently not enough evidence to indicate one over another for patients with BSS. We discussed the mode of delivery with the patient and she manifested preference for c-section. She declared having no longer reproductive desire, so that the c-section risks of complicating a future pregnancy were not taken as relevant. General anesthesia is superior to regional anesthesia, because of the risk of bulky epidural hematoma [2,10].

Absence of complications during ante- and peripartum periods does not predict how the woman will do subsequently. Strict vigilance is warranted at least until six weeks postpartum, due to the virtual risk of secondary postpartum hemorrhage [7,8]. Our patient was advised to immediately contact our maternity hospital whether she detected any symptom compatible with ongoing bleeding on the next weeks. Her late postpartum period was uneventful, though.

It is of major relevance to educate patients with lifelong bleeding disorders on how to identify a clinically significant bleeding and when to seek medical attention for it [1]. Prevention of trauma and correct treatment of diseases that may induce bleeding enable the patient to have a fairly normal life [3]. Also, patient’s doubts and fears must not be neglected. In the process of coping with the disease, a supporting multidisciplinary team to treat the patient globally is invaluable.

Thereby, we focused on ensuring that our patient would not be managed only with the obstetrical outcome in mind. She was taught general bleeding-preventive measures, such as avoiding the use of aspirin and other antiplatelet drugs [1]. Avoidance of contact sports was also recommended. In face of her complaint of frequent mild gum bleeding, she was assessed by a dentist and was counseled about adequate oral hygiene. Gingivitis is one of the most prevalent periodontal problems worldwide, and commonly manifests with spontaneous bleeding. On individuals with increased bleeding diathesis, however, it may represent a problem of greater magnitude, and must therefore not be overlooked. After being educated about key points regarding her condition, our patient was discharged.

## Conclusions

BSS is a rare bleeding disorder that may complicate pregnancy. Pregnancy course of women affected by the syndrome is widely variable and, to some extent, unpredictable. Strict vigilance of the mother’s hematocrit and platelet count is advised, thus, in order to readily diagnose and allow the correction of any ongoing bleeding in the peripartum period.

## Consent

Written informed consent was obtained from the patient for publication of this case report. A copy of the written consent is available for review by the Editor-in-Chief of this journal.

## Abbreviations

BSS: Bernard-Soulier syndrome
CBC: Complete blood count
Hb: Hemoglobin
Hct: Hematocrit
PC: Platelet count
